# Capric Acid Secreted by *S. boulardii* Inhibits *C. albicans* Filamentous Growth, Adhesion and Biofilm Formation

**DOI:** 10.1371/journal.pone.0012050

**Published:** 2010-08-10

**Authors:** Anna Murzyn, Anna Krasowska, Piotr Stefanowicz, Dorota Dziadkowiec, Marcin Łukaszewicz

**Affiliations:** 1 Faculty of Biotechnology, University of Wrocław, Wrocław, Poland; 2 Faculty of Chemistry, University of Wrocław, Wrocław, Poland; 3 Faculty of Chemistry, Wrocław University of Technology, Wrocław, Poland; Massachusetts General Hospital, United States of America

## Abstract

Candidiasis are life-threatening systemic fungal diseases, especially of gastro intestinal track, skin and mucous membranes lining various body cavities like the nostrils, the mouth, the lips, the eyelids, the ears or the genital area. Due to increasing resistance of candidiasis to existing drugs, it is very important to look for new strategies helping the treatment of such fungal diseases. One promising strategy is the use of the probiotic microorganisms, which when administered in adequate amounts confer a health benefit. Such a probiotic microorganism is yeast *Saccharomyces boulardii*, a close relative of baker yeast. *Saccharomyces boulardii* cells and their extract affect the virulence factors of the important human fungal pathogen *C. albicans*, its hyphae formation, adhesion and biofilm development. Extract prepared from *S. boulardii* culture filtrate was fractionated and GC-MS analysis showed that the active fraction contained, apart from 2-phenylethanol, caproic, caprylic and capric acid whose presence was confirmed by ESI-MS analysis. Biological activity was tested on *C. albicans* using extract and pure identified compounds. Our study demonstrated that this probiotic yeast secretes into the medium active compounds reducing candidal virulence factors. The chief compound inhibiting filamentous *C. albicans* growth comparably to *S. boulardii* extract was capric acid, which is thus responsible for inhibition of hyphae formation. It also reduced candidal adhesion and biofilm formation, though three times less than the extract, which thus contains other factors suppressing *C. albicans* adherence. The expression profile of selected genes associated with *C. albicans* virulence by real-time PCR showed a reduced expression of *HWP1*, *INO1* and *CSH1* genes in *C. albicans* cells treated with capric acid and *S. boulardii* extract. Hence capric acid secreted by *S. boulardii* is responsible for inhibition of *C. albicans* filamentation and partially also adhesion and biofilm formation.

## Introduction

*Saccharomyces boulardii* (Biocodex, Gentilly, France) is a nonpathogenic yeast used as a probiotic strain in prevention or treatment of intestinal diseases, mainly different types of diarrhea [1], [2], [3], [4]. The activity of *S. boulardii* against enteric pathogens involves many different mechanisms and can be caused either by *S. boulardii* cells or by agents secreted by them, such as the 120 kDa protein which inhibits cholera toxin-induced adenylate cyclase and chloride secretion [5] or the 63 kDa phosphatase that exerts dephosphorylation activity against LPS of entheropathogenic *E. coli* [6], and a 54 kDa proteinase which degrades both *Clostridium difficile* toxins A and B [7]. Moreover, *S. boulardii* stimulates the activity of brush-border membrane enzymes implicated in nutrient degradation and absorption possibly by the secreting polyamines [8]. There are many investigations describing the effect of *S. boulardii* on bacterial pathogens, but little is know about its influence on yeast pathogen *Candida albicans*.

*C. albicans* is the most common opportunistic fungal pathogen isolated from human body, causing both superficial and systemic diseases. Infections develop often after antibiotic treatment, the most serious being prevalent in immunocompromised patients [9]. The major virulence factor of *C. albicans* is the capacity to switch between yeast, pseudohyphae and hyphae, important both for tissue adhesion and invasion [10]. *C. albicans* strains locked into either the yeast or filamentous form are less virulent [11], [12]. *C. albicans* virulence also depends on its ability to adhere to and form biofilms on many surfaces, such as medical implants, intravascular catheters, or host tissues [13], [14] as well as secretion of enzymes like secreted aspartyl proteinases and phospholipases that play important role in tissue invasion [15], [16].

Increasing resistance of *C. albicans* strains to antifungal agents prompted higher interest in molecular mechanisms of its virulence in search for new targets for therapy. Factors involved in hyphal development may constitute such targets and agents inhibiting formation of hyphae can lead to great improvements in antifungal therapy. It is known that farnesol, a quorum sensing molecule secreted by *C. albicans* itself, blocks the conversion of its yeast cells to hyphae. Isoamyl alcohol, 2-phenylethanol and 1-dodecanol are other compounds known to control morphological transformation of *C. albicans* [17]. Undecylenic acid, which belongs to the group of fatty acids, has also been found to have similar activity [18]. Fatty acids or their monoglyceride derivatives have long been known as antimicrobial agents that kill Gram-positive and Gram-negative bacteria. They also exhibit antiviral and antifungal activity. Studies of Kabara et al. (1972)[19] revealed that lauric acid with a 12 carbon chain is the most active among saturated fatty acids. Ells et al. (2009) [20] reported that arachidonic acid increased antifungal susceptibility of *C. albicans* biofilm. Furthermore, capric acid and lauric acid were found to effectively kill *C. albicans* cells [21]. Clément et al. (2006) [22] demonstrated the suppression of candidal hyphae formation by whey-derived fatty acids, among which the most active were lauric acid, myristoleic acid, linoleic acid and arachidonic acid.

Recently, it was shown that *S. boulardii* decreases inflammatory reaction and colonization of mouse intestine by the *C. albicans* infection [23]. Moreover, intraepithelial lymphocytes infected by *E. coli* and *C. albicans* were found to respond to the presence of *S. boulardii* by decreasing IL-1β secretion [24]. Translocation of *C. albicans* from the intestinal tract to the mesenteric lymph nodes (MLN) and some organs was reduced after treatment with *S. boulardii* [25].

We have previously shown that the presence of *S. boulardii* cells and an extract from its culture filtrate inhibited *C. albicans* hyphae formation, adhesion and biofilm formation on plastic surfaces [26]. The present study was focused on the identification of factors secreted by *S. boulardii* - fatty acids and 2-phenylethanol - and analysis of their activities. We demonstrated the influence of these factors on *C. albicans* ability to form hyphae and adhere to plastic surfaces and examined which *C. albicans* genes, associated with its virulence, are regulated by the exposure to capric acid (C10:0).

## Results and Discussion

We have previously shown that the presence of *S. boulardii* cells inhibited yeast to hyphae switch of *C. albicans*. *S. boulardii* also reduced *C. albicans* adhesion and subsequent biofilm formation on plastic surfaces [26]. Not only *S. boulardii* cells were active, but also an extract prepared from its culture filtrate exerted the same effect against *C. albicans*, suggesting that *S. boulardii* secretes into the medium active factor/s which are efficiently extracted into ethyl acetate.

### Determination of chemical structure of compounds secreted by *S. boulardii*

**Figure 1:**
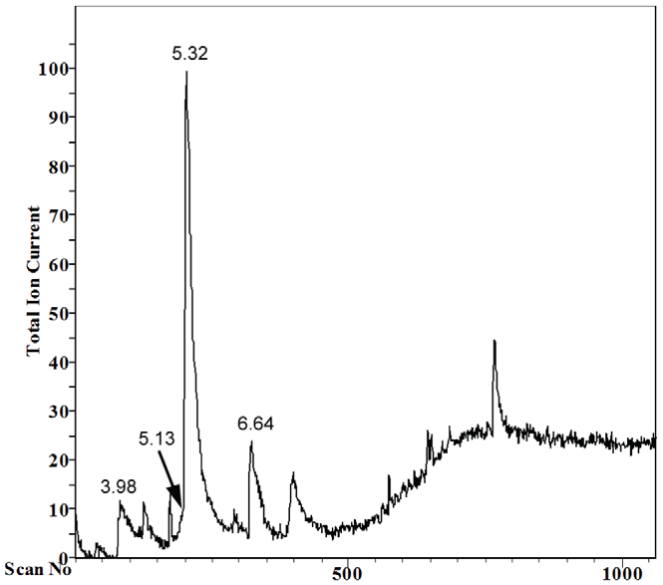
GC-MS analysis of active fraction separated from *S. boulardii* extract. Peak at 3.98 min corresponds to presence of caproic acid (C6:0), peak at 5.13 min to 2-phenylethanol, peak at 5.32 min to caprylic acid (C8:0), peak at 6.64 min to capric acid (C10:0).

In order to identify the active compound/s *S. boulardii* extract was first partially separated using preparative TLC. The seven collected fractions were then checked for the biological activity in filamentation assay in RPMI-1640 medium. Hyphae formation was inhibited by one fraction. This partially purified fraction was thus analyzed by GC-MS. GC showed several peaks (Fig. 1) which were further examined by MS. The analysis of the results using data system library (NIST 49 K) indicated the presence of short chain fatty acids: caproic acid (C6:0) at 3.98 min peak, caprylic acid (C8:0) at 5.32 min peak and capric acid (C10:0) at 6.64 min peak. Additionally, peak at 5.13 min was identified as 2-phenylethanol.

Subsequently, commercially available standards of these fatty acids and 2-phenylethanol were compared with the active fraction for their retention times and mass spectra. Commercial compounds exhibited similar retention times and the same fragmentation patterns as compounds found in the *S. boulardii* extract, confirming the presence of caproic acid (6:0), caprylic acid (8:0), capric acid (10:0) and 2-phenylethanol in its active fraction (Fig. S1).

**Figure 2:**
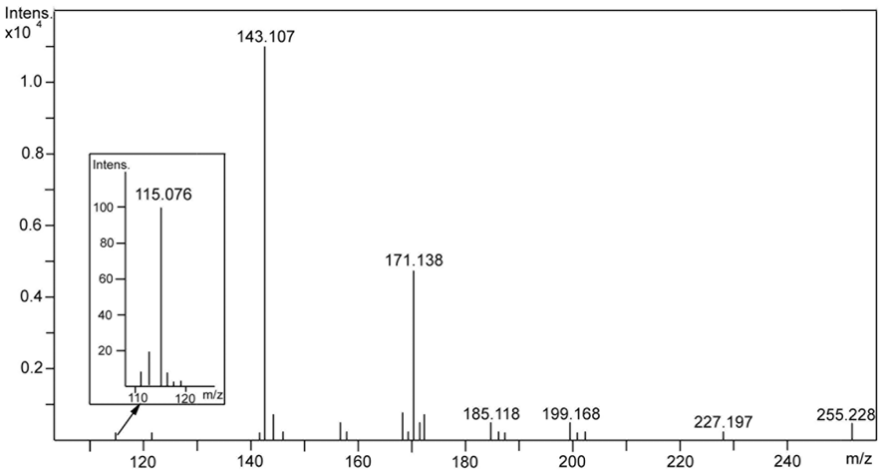
ESI-MS pattern of active fraction showing ions at *m/z* 115.076, 143.107 and 171.138.

To further confirm the identification of compounds, the active fraction was analyzed by ESI-MS system, which provides a minimal fragmentation of the analyzed sample during ionization. The negative ionisation analysis showed the appearance of molecular ions at *m/z* 115.076, 143.107 and 171.138 (Fig. 2). The broader *m/z* window 100–1000 *m/z* is included in Fig. S2. The *m/z* values for detected ions were the same as calulated from the formulas of monoisotopic caproic (C6:0), caprylic (C8:0) and capric (C10:0) acids ions [M–H]^−^ which are 115.076, 143.107 and 171.139, respectively. The relatively low abundance of peak at *m/z* 115.076 results not only from the low concentration of caproic acid (C6:0) in the sample but also from a decrease in sensitivity of the Bruker micrOTOF-Q spectrometer in the range of *m/z* close to 100 Da. Additionally ESI-MS spectra were obtained for standards of caprilic (Fig. S3) and capric acids (Fig. S4). The peaks of [M–H]- ions were observed in both cases and the fragmentation in ion source was not detected. 2-Phenylethanol was not detected in ESI-MS analysis, because ionization of this compound in ESI experiment is poor.

The result confirmed the presence of three fatty acids with 6-, 8- and 10-carbon in chain length in the active fraction.

*S. boulardii* extract and its active fraction were screened for the presence of trans,trans-farnesol, which has been previously shown to have similar activity as the compounds secreted by *S. boulardii*. However, this molecule was not detected by GC-MS analysis; this is consistent with our previous UPLC based data that farnesol could not be responsible for *S. boulardii* extract activity [26]. At the first sight there are structural similarities of capric acid with farnesol having common biological activities (e.i. reduced hyphae formation and repression of *HWP1*). In the same time there are substantial differences in the chemical character. As we show, two carbon difference in aliphatic chain length may significantly influence biological activity. Carboxylic acid is much more amphiphatic than farnesol with hydroxyl group. Finally, saturated aliphatic chain is less rigid than unsaturated found in farnesol. Basing on the biological activity and chemical structure it is difficult to judge if the molecular mechanisms of action are common.

**Figure 3:**
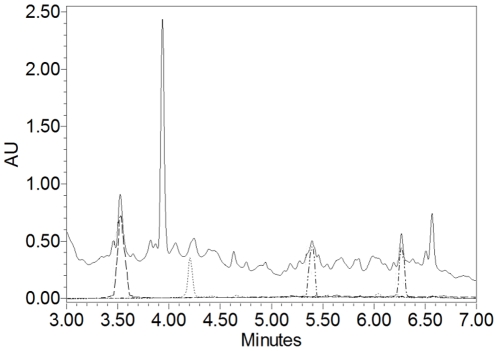
UPLC chromatograms of 2-phenylethanol and fatty acids as compared with *S. boulardii* extract monitored at 220 nm. Solid line represents 96 µg of *S. boulardii* extract, dashed line 5.1 µg of 2-phenylethanol, dotted line 17.4 µg of caproic acid (C6:0), dash-dot-dot line 21.4 µg of caprylic acid (C8:0), dash-dot line 25.8 µg of capric acid (C10:0).

To calculate the amounts of fatty acids and 2-phenylethanol in the extract, the area of UPLC chromatogram peak of known concentration of each commercially available pure standards was compared with corresponding peaks present in *S. boulardii* extract. UPLC analysis of extract showed peaks consistent with the retention time of peaks for indicated compounds (Fig. 3), additionally confirming that these compounds are secreated by *S. boulardii*. Calculation revealed that 160 µg of *S. boulardii* extract contains at the most 37.8 µg of caproic acid (C6:0), 17.9 µg of caprylic acid (C8:0), 45.3 µg of capric acid (C10:0) and 4.2 µg of 2-phenylethanol. These concentrations of tested compounds were used in further experiments.

Capric acid (10:0) has been shown to kill *C. albicans* at very high concentration (10 mM = 1720 µg/ml) however it was able to inhibit fungal growth at a concentration of 2.5 mM (430 µg/ml) [21]. To exclude the possible killing effect on fungal cells by the concentrations of identified fatty acids used in our study, the viability test was performed. None of the tested compounds alone inhibited *C. albicans* growth at concentrations which are present in 160 µg/ml of *S. boulardii* extract (the highest amount used in the experiments).

### Effect of the compounds on *C. albicans* filamentation

In order to determine whether the free fatty acids and 2-phenylethanol are responsible for the inhibition of *C. albicans* hyphae formation, the activity of each pure compound was compared with the activity of *S. boulardii* extract. It is commonly known that acidic pH can strongly inhibit filamentation of *C. albicans* [27]. Fatty acids used in biological activity assay could lower the pH of the medium so each sample was checked for the pH value before the experiments. It was found that the pH of RPMI-1640 (pH 7.5) was not affected by the addition of each compound at tested concentrations.

**Figure 4:**
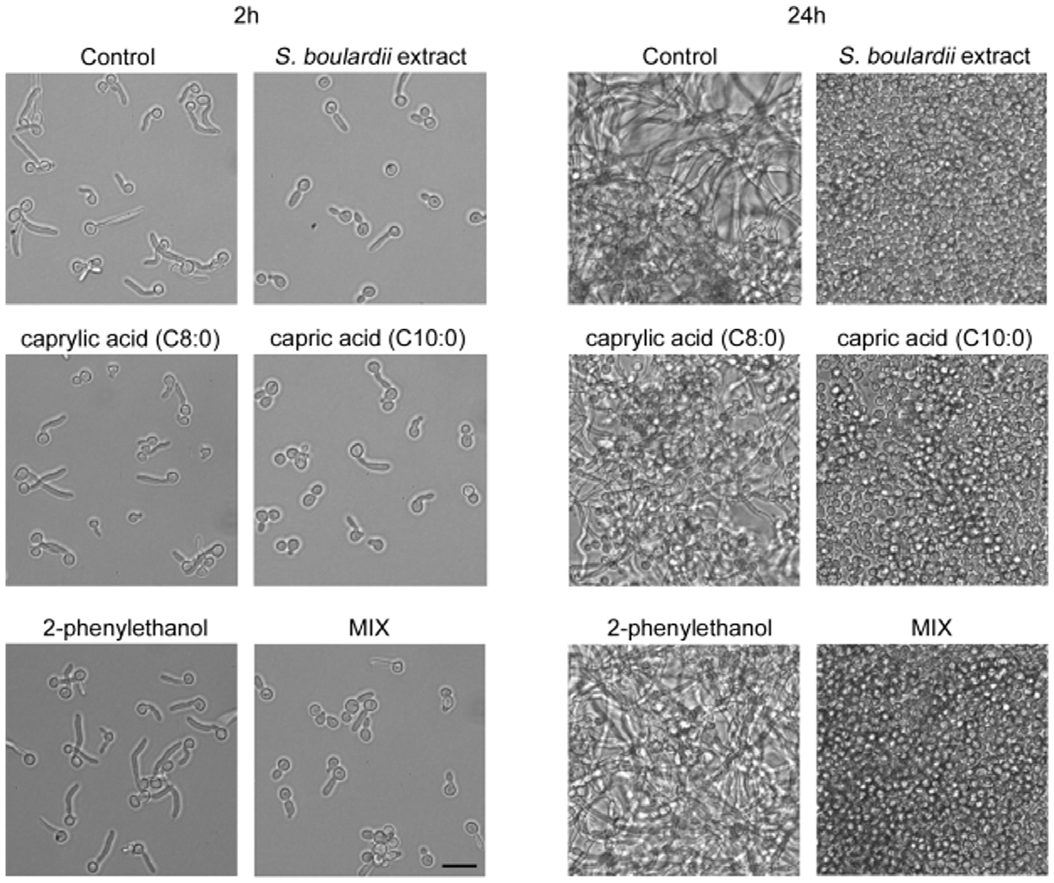
*C. albicans* morphology incubated for 2 h (left panel) and 24 h (right panel) in RPMI-1640 at 37°C in the presence of the compounds. Control sample contained 1% methanol, other samples contained 160 µg/ml of *S. boulardii* extract, 17.9 µg/ml of caprylic acid (C8:0), 45.3 µg/ml of capric acid (C10:0) or 4.2 µg/ml of 2-phenylethanol. MIX – mixture of all the compounds at concentrations indicated above. Bar, 10 µm.

Under the tested conditions, the capric acid (C10:0) alone showed an inhibitory activity against hyphae formation comparable to the *S. boulardii* extract alone (Fig. 4, left panel), as shown by ratio of cells in yeast form compared to that with formed germ tubes after 2 h of incubation. Caprylic acid (C8:0) had only a slight effect on filamentation while caproic acid (C6:0) did not exhibit any inhibitory action against *C. albicans* germ tube formation (data not shown).

Inhibition of hyphae formation in *C. albicans* was previously shown by fatty acids isolated from whey cream, mainly lauric acid, myristoleic acid, palmitoleic acid, linoleic acid and arachidonic acid [22]. The authors claimed that also capric acid (C10:0) was effective, though they did not isolate it.

2-Phenylethanol was not found to affect the yeast-to-hyphae switch even at concentrations which were previously reported to inhibit *C. albicans* morphological transition [17]. This discrepancy may be due to a different experimental model of hyphae formation assay or to different susceptibility of *C. albicans* strains used in study. A mixture of all tested compounds did not show stronger effect than capric acid (C10:0) alone. This suggests that there is no synergy between the tested molecules and capric acid (C10:0) is responsible for the observed inhibition of *C. albicans* filamentation.

After 24 h of incubation in the presence of tested compounds at concentrations that do not affect cell growth, *C. albicans* reached similar density in all samples (Fig. 4, right panel). While control and 2-phenylethanol-treated culture contained a dense network of long filaments, principally only yeast forms of *C. albicans* were present in samples with *S. boulardii* extract, capric acid (C10:0) and mixture of all compounds. Caprylic acid (C8:0) treated sample contained some long filaments with considerable number of the cells existing in yeast form pointing to a weaker activity of this acid.

### Effect of the compounds on *C. albicans* adhesion and biofilm formation

**Figure 5:**
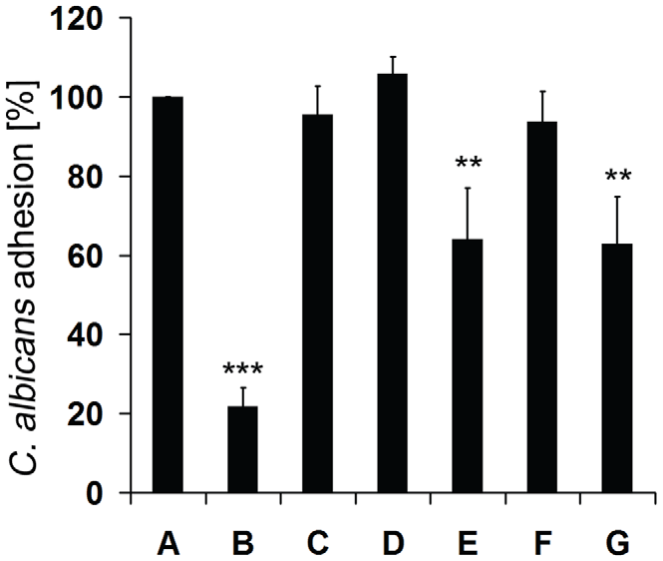
Effect of the compounds on *C. albicans* adhesion. (A) control sample with 1% methanol, (B) 160 µg/ml of *S. boulardii* extract, (C) 37.8 µg/ml of caproic acid (C6:0), (D) 17.9 µg/ml of caprylic acid (C8:0), (E) 45.3 µg/ml of capric acid (C10:0), (F) 4.2 µg/ml of 2-phenylethanol, (G) mixture of all these compounds at concentrations indicated above. Statistical analysis was performed using paired Student *t*-test. *P* values<0.05 were considered significant. Double stars 0.001<*P*<0.01, triple stars *P<*0.001.

To determine if the fatty acids and 2-phenylethanol are also responsible for the reduction of *C. albicans* adherence, commercial compounds were assayed in adhesion test and compared with *S. boulardii* extract activity. The extract reduced the ability of *C. albicans* cells to adhere by about 78% (Fig. 5 B) while capric acid (C10:0) caused only 36% reduction (Fig. 5 E), similar to that found in the sample treated with mixture of all tested compounds (Fig. 5 G). This result indicates that the extract contains, apart from capric acid (C10:0), other as yet unidentified molecule/s which inhibit *C. albicans* adherence. Caproic acid (C6:0), caprylic acid (C8:0) and 2-phenylethanol did not have any effect on *C. albicans* adhesion (Fig. 5 C, D, F).

Adhesion subsequently leads to biofilm formation. Since capric acid (C10:0) inhibited adhesion it could also reduce biofilm formation. Indeed, the inhibitory effect of capric acid (C10:0) was maintained after 48 h of incubation. The level of biofilm in *C. albicans* sample treated with this fatty acid reached only 0.74±0.16 arbitrary OD units, while values for control sample reached 2.54±0.10, as measured by the intensity of crystal violet staining. *S. boulardii* extract reduced biofilm formation still more - to OD 0.17±0.04 (data not shown).

### Effect of capric acid on *C. albicans* gene expression

Since the most active molecule produced by *S. boulardii* inhibiting filamentation and partially also *C. albicans* adhesion is capric acid (C10:0), we analyzed changes of chosen gene expression levels in *C. albicans* cells treated with capric acid (C10:0) and *S. boulardii* extract. The genes were chosen for their implication in virulence of *C. albicans*, like hyphae formation (*HWP1*, *EFG1*, *HST7*, *CRK1*, *BIG1*), adhesion process (*HWP1*, *EFG1*, *CSH1*, *BIG1*), maintenance of cell wall integrity during growth and morphogenesis (*CHT3*) or other features involved in its pathogenicity (*INO1*).

**Figure 6:**
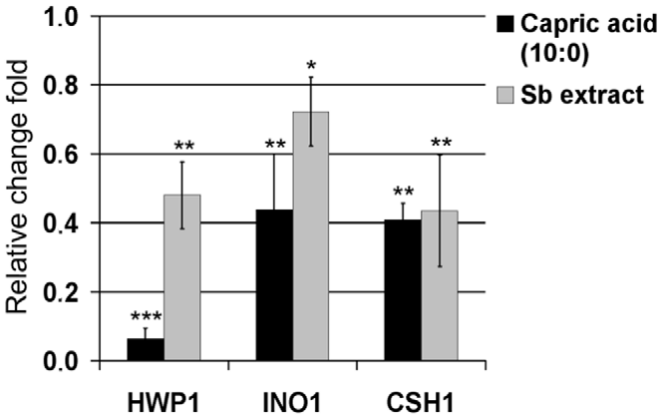
Relative expression of 3 mRNAs of *C. albicans* cells treated with 45.3 µg/ml of capric acid (C10:0) and 160 µg/ml of *S. boulardii* extract. Statistical analysis was performed using one sample Student *t*-test. *P* values<0.05 were considered significant. Single stars denote 0.01<*P*<0.05, double stars 0.001<*P*<0.01, triple stars *P<*0.001.

The expression of genes is shown as relative values referred to the untreated control that was set to one. Significant changes in expression were found for three genes (Fig. 6). Both *S. boulardii* extract and capric acid (C10:0) decreased the expression of *HWP1* gene encoding a hyphal wall protein involved in adhesion and biofilm formation [28], [29]. This corresponded very well to observed changes in morphology and adherence of *C. albicans* cells treated with the two agents. Capric acid (C10:0) reduced *HWP1* transcript level 8 times more than *S. boulardii* extract, which contains many other compounds (UPLC, GCMS data, not shown) that may have an effect opposite to that of capric acid (C10:0). Decreased expression of hyphae specific gene *HWP1* was also previously detected after treatment with farnesol [30]. *HWP1* is a downstream component of cAMP-PKA dependent pathway and is positively regulated by *EFG1*, but its expression is independent of the MAP kinase cascade, the second well-known pathway implicated in hyphae formation [11], [31]. Since capric acid (C10:0) decreased the expression of *HWP1*, it is possible that it inhibits the Efg1-dependent gene induction pathway. However, *HWP1* expression is also controled by *TUP1* and *RBF1*, negative and positive regulators of hyphal development, respectively [12], [31].

*C. albicans* cells treated with *S. boulardii* extract and capric acid (C10:0) showed a reduced level of *INO1* (Fig. 6), which is required for the synthesis of phospholipomannan, a GPI-anchored glycolipid on *C. albicans* cell surface important for its virulence [32]. As previously reported, *INO1* was upregulated in drug-resistant *C. albicans* strains [33] and farnesol strongly decreased its expression in *C. albicans* biofilm [34].

Exposure to both *S. boulardii* extract and capric acid (C10:0) also reduced the expression of *CSH1* that encodes a protein associated with the cell surface hydrophobicity and believed to be involved in *C. albicans* virulence, including inhibition of adhesion to fibronectin [35], [36]. Decreased expression of *CSH1* is consistent with the observed inhibitory effect on adhesion and biofilm formation by *C. albicans* (Fig. 6). Reduction of *CSH1* expression was also previously found for farnesol treated biofilm of *C. albicans* [34].

The expression of other tested genes was not significantly changed after exposure to *S. boulardii* extract.

In conclusion, the results presented herein provide new evidence that *S. boulardii* secretes into the medium fatty acids and 2-phenylethanol, among which capric acid (C10:0) is the most effective in inhibiting essential virulence factors of *C. albicans*, especially morphological transition and partly adhesion as well as biofilm formation. Capric acid (C10:0) also reduced expression of *HWP1*, *INO1* and *CSH1* genes related to *C. albicans* pathogenicity. Although capric acid (C10:0) is a major player reducing morphological transformation, there is a clear difference in biological *C. albicans* response after treatment with pure compound and extract from *S. boulardii*. Thus, to explain myriad of benefits coming from *S. boulardii* administration, it is necessary to explore the activity and interactions of other compounds secreted by this organism.

## Materials and Methods

### Strains and growth conditions

*C. albicans* strain SC5314 [37] was used in this study. Lyophilized *S. boulardii* culture was supplied by Biocodex, Ultra-Levure® (Gentilly, France). An isolated colony of each strain was inoculated into 5 ml of Yeast Nitrogen Base broth (YNB pH 5.5) (Difco Laboratories, Detroit, Mich.) containing 2% D-glucose, and incubated overnight at 30°C. One hundred µl of such pre-culture was inoculated into 5 ml of YNB, incubated for 24 h at 30°C and used for all experiments.

### Preparation of *S. boulardii* extract

*S. boulardii* extract was prepared from culture filtrate as described previously [26]. Briefly, two liters of 24 h culture of *S. boulardii* were centrifuged at 3.000×g for 10 min. The supernatant was decanted and extracted with one-fifth volume of ethyl acetate with changes of ethyl acetate every half an hour of extraction. The ethyl acetate was removed on a rotary evaporator and the residue was resuspended in methanol to give the final concentration of 48 mg/ml of residue in the stock sample. The extract was diluted to give the final concentration of 160 µg/ml in each sample. The concentration of methanol in a sample was never >1%.

### Fractionation of *S. boulardii* extract

The *S. boulardii* extract was fractionated on a silica plate containing a fluorescent indicator with glass bottom (20 cm×20 cm×0.2 cm, Macherey Nagel, Düren, Germany) using a chloroform/methanol (20∶1, v/v) mobile phase in a vertical glass chamber. Then the plate was air dried and observed under 254 nm. The silica on the plate was divided into 7 fractions by UV active bands and scraped into separate tubes. It was subsequently washed 3 times with methanol. The volume of collected methanol from each fraction was reduced using rotary evaporator (Heidolph, Schwabach, Germany) to concentrate samples and analyzed for the biological activity in hyphae formation assay and by GC-MS and ESI-MS analysis for the determination of chemical structures of relevant compounds.

### GC-MS analysis

Active fraction and *S. boulardii* extract was analysed by Hewlett-Packard 5890 II coupled with Hewlett-Packard HP-5971 A (Hewlett-Packard, Palo Alto, CA) mass selective detector with Elite 5 MS column (25 m×0.2 mm×0.33 µm) in a splitless mode. The oven temperature was programmed from 70 to 290°C and the transfer line was heated at 280°C. Helium carrier gas had a flow of 1 ml/min. The mass spectrometer was operated in the electron impact mode at 70 eV, scanning the range of 35–550 *m/z* in a full scan acquisition mode. The identification of compounds was achieved by comparing the GC retention times and mass spectra with those of pure of caproic acid (C6:0), caprylic acid (C8:0), capric acid (C10:0) and 2-phenylethanol standards (Sigma-Aldrich, St. Louis, Mo). All mass spectra were also compared with the data system library (NIST 49 K).

### ESI-MS analysis

High-resolution ESI-MS spectra were obtained on a Bruker micrOTOF-Q spectrometer (Bruker Daltonik, Bremen, Germany), equipped with Apollo II electrospray ionization source with ion funnel, operated in the negative ion mode. The sample in methanol (0.05 mg/ml) was infused at a flow rate of 3 µl/min. The potential between the spray needle and the orifice was set to 4.5 kV. Before each run the instrument was calibrated externally with the Tunemix™ mixture (Bruker Daltonik, Bremen, Germany) in quadratic regression mode.

### Ultra performance liquid chromatography (UPLC)

UPLC analysis was performed using a Waters ACQUITY UPLC Systems with a PDA detector with the column (length 5 cm with 1.7 small-particle chemistries; Waters Corp., Milford, Mass.). Commercial fatty acids and 2-phenylethanol (Sigma-Aldrich) were diluted in methanol. 17.4 µg of caproic acid (C6:0), 21.4 µg of caprylic acid (C8:0), 25.8 µg of capric acid (C10:0), 5.1 µg of 2-phenylethanol and 96 µg of *S. boulardii* extract were injected into UPLC column. The elution was carried out with methanol gradient, starting with 10% methanol and reaching 100% in 8 min and being run for another 4 min. The chromatograms of all standard compounds were monitored at 220 nm and compared with the chromatogram obtained for *S. boulardii* extract. The amount of fatty acids and 2-phenylethanol in *S. boulardii* extract was calculated by comparing areas under corresponding chromatogram peaks.

### Hyphae formation assay

Two µl of *C. albicans* culture was added to every 0.5 ml of RPMI-1640 medium, containing 160 µg/ml of *S. boulardii* extract or one of the *S. boulardii* fractions isolated by TLC plate fractionation. In different experiment the 37.8 µg/ml caproic acid, 17.9 µg/ml of caprolic acid, 45.3 µg/ml of capric acid and 4.2 µg/ml 2-phenylethanol (Sigma-Aldrich) were used. All diluted in methanol; the concentration of methanol in a sample was never >1%. As a control, RPMI-1640 with 1% of methanol was used. The assay was carried out in a 24-well plate (Nunc, Roskilde, Denmark). After 2 and 24 h of incubation at 37°C the wells were examined under an inverted microscope Olympus CKX41 and photographed using the ARTCAM-300MI camera (Olympus, Tokyo, Japan) with 40× magnification. Inactive fractions or control contained 2–5% of yeast cells, while active fractions contained 50–100% of yeast cells.

### Adhesion assay

*C. albicans* adhesion assay was performed as described previously (Krasowska et al., 2009). Briefly, 50 µl of *C. albicans* culture (5×10^6^ cells/ml) and 50 µl of RPMI-1640 were added to each well together with the appropriate amount of *S. boulardii* extract and fatty acids or 2-phenylethanol. The wells containing *C. albicans* culture only in RPMI-1640 with 1% methanol were used as control. After 1 hour incubation at 37°C nonadherent cells were removed by three washes with phosphate buffer saline pH 7.4. Adherent cells were stained with 0.1% crystal-violet for 5 min and again washed three times with phosphate buffer saline pH 7.4. Next, 150 µl of isopropanol-0.04 M HCl and 50 µl of 0.25% SDS were added to each well to resolubilize crystal violet. The absorbance of each well was measured using a microplate reader UVM340 at A_590_ (Asys Hitech GmbH, Eugendorf, Austria). Assays were carried out three times in five replicates.

In case of biofilm formation, the experiment was performed as described above, but the plate was incubated for 48 h at 37°C.

### Gene expression assay

After 24 h incubation at 30°C *C. albicans* cells were harvested, washed with phosphate buffer saline and diluted in 18 ml RPMI-1640 medium to give a final concentration of 5×10^6^ cells/ml. Samples contained 6 ml of such *C. albicans* culture and 160 µg/ml of *S. boulardii* extract or 45.3 µg/ml capric acid (C10:0) or only 1% methanol for control. All samples were incubated at 37°C for 2 h and then centrifuged at 1000×g for 5 min. Total RNA was isolated from obtained pellets using Total RNA Mini isolation kit (A&A Biotechnology, Poland) following manufacturer's instructions. Subsequently, the isolated RNA was digested with DNase I, RNase-free (Fermentas International, Burlington, Canada). cDNA synthesis was performed using High-Capacity cDNA Reverse Transcription Kit (Applied Biosystems, Foster City, CA) following manufacturer's instructions. Primers for real-time PCR were designed using LightCycler Probe Design Software 2.0 (Table 1). Actin gene was used as endogenous control. The relative concentration of each transcript was determined using RealTime 2xPCR Master Mix SYBR B (A&A Biotechnology, Gdansk, Poland) on Light Cycler 2.0 (Roche, Indianapolis, IN). Each PCR protocol consisted of a primary denaturation step at 95°C for 20 sec, followed by 30 cycles of denaturation at 95°C for 20 sec, annealing at 45°C and extension at 72°C for 15 sec. Each product was checked for its specificity in melting curve analyses and efficiency of the amplification was verified with standard curves for every gene. Results were analyzed by LightCycler Software 4.0. Each assay was repeated three times for separately isolated RNA.

**Table 1:** Sequences of primers used for RT-PCR.

	Forward primer	Reverse primer
ACT1	TTTAAGAATTGATTTGGCT	GAAGATTGAGAAGAAGTTT
BIG1	TTATTCGTCCTACTAGCAT	CATATTTGTCACCGAAGTAA
CHT3	GTATTTCCAAATCCAGTTC	GTCAATATTTGATAAGTCG
CRK1	CAGGTGGAATGGATACTTA	GTACTATTTGAAGTCAACG
CSH1	AAATGGTAAATCTGAAGAG	CCAATATATCTTGCCAATC
HST7	TCATCAGCTTCTTCTATAC	TATTGAGGAAATGACAGTT
HWP1	ATTCCAAATATTCCAACTG	TTCTGAAGTGGTAGCTAAA
INO1	TTCAGAATTAATGTTGGGT	TTCTTTCAAATCTTAATTCGT
EFG1	GCCTTATAACTACATGTTC	TGGTAATAGTTTCCTTGAG

### Statistical analysis

Statistical analysis was performed using paired Student *t*-test, with Bonferroni correction or one sample Student *t*-test. *P* values <0.05 were considered significant. Single stars denote 0.01<*P*<0.05, double stars 0.001<*P*<0.01, triple stars *P<*0.001.

## Supporting Information

Figure S1 The ESI/MS spectrum of active fraction in 100-1000 m/z window.
(0.27 MB PNG)

Figure S2 The comparison of fragmentation pattern and retention times of commercially available standards with compounds being in active fraction.
(0.36 MB PNG)

Figure S3 ESI/MS spectrum of caprylic acid (C8:0) standard.
(0.36 MB PNG)

Figure S4 ESI/MS spectrum of capric acid (C10:0) standard.
(0.31 MB PNG)
