# Supplementary figures and images for: Capric Acid Secreted by S. boulardii Inhibits C. albicans Filamentous Growth, Adhesion and Biofilm Formation
Source: PLoS One. 2010 Aug 10;5(8):e12050. doi: 10.1371/journal.pone.0012050 (PMC2919387; doi:10.1371/journal.pone.0012050)

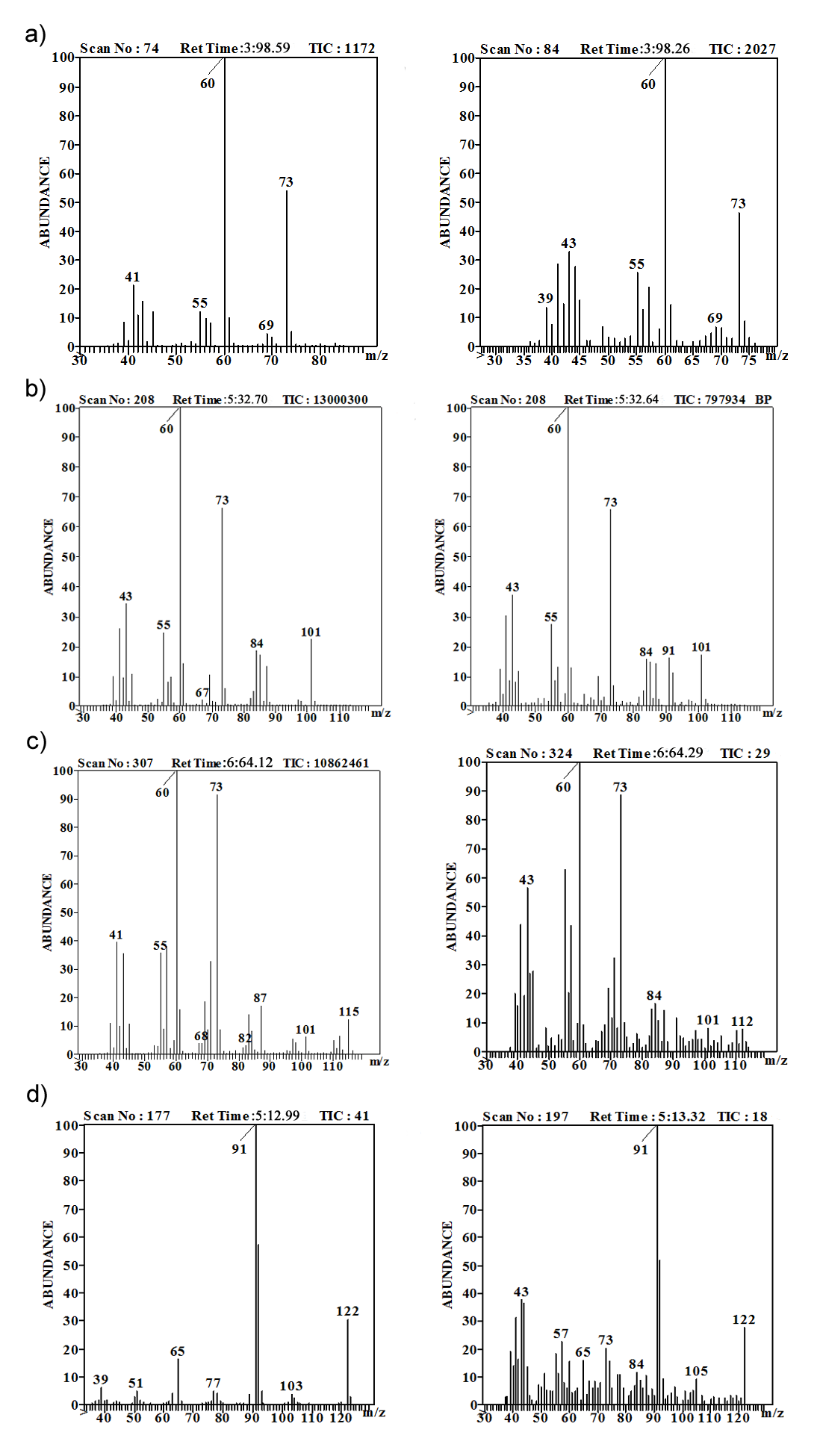

Supplement: Figure S1 — The ESI/MS spectrum of active fraction in 100-1000 m/z window. (0.27 MB PNG) [file pone.0012050.s001.png]

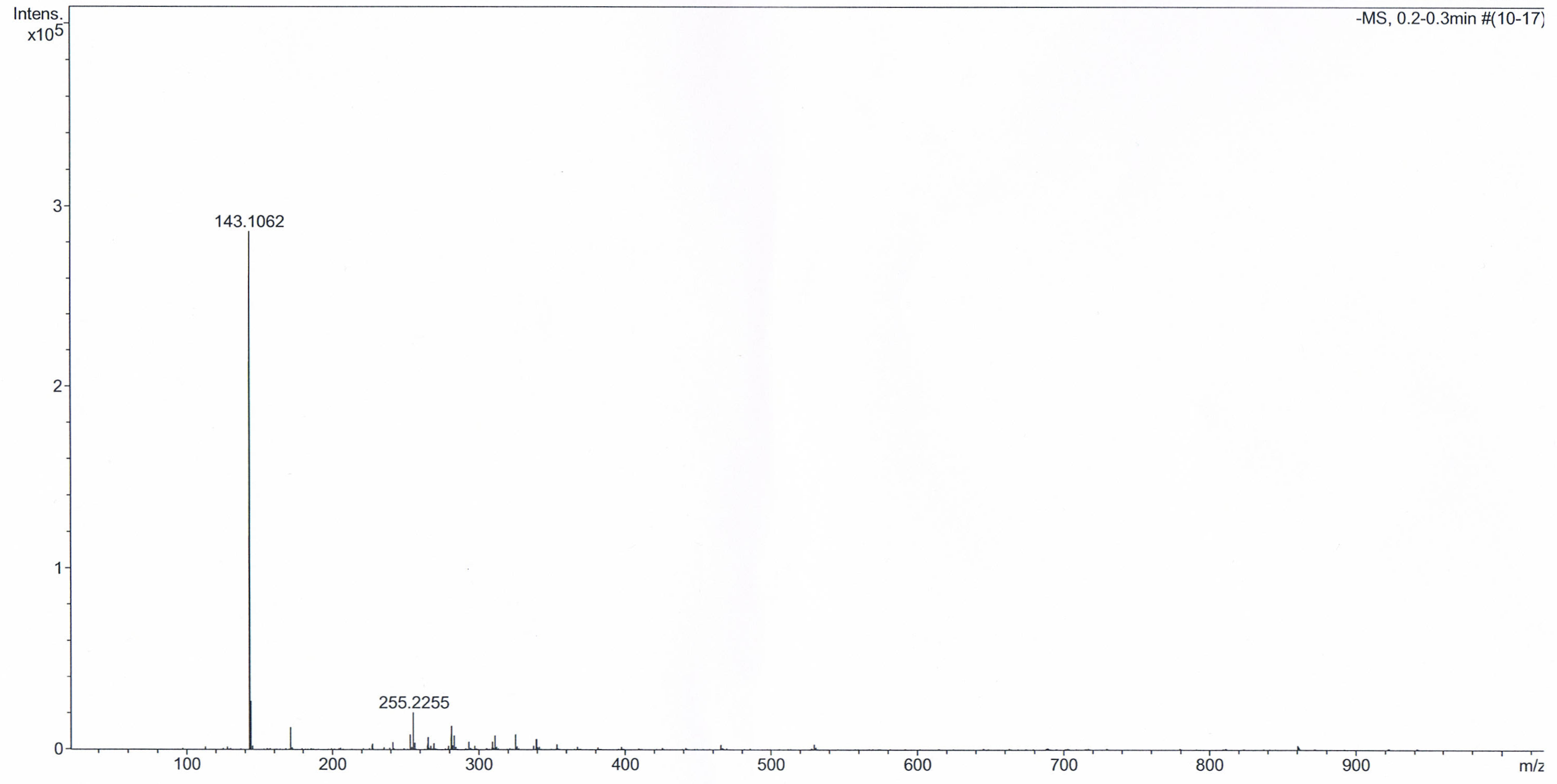

Supplement: Figure S2 — The comparison of fragmentation pattern and retention times of commercially available standards with compounds being in active fraction. (0.36 MB PNG) [file pone.0012050.s002.png]

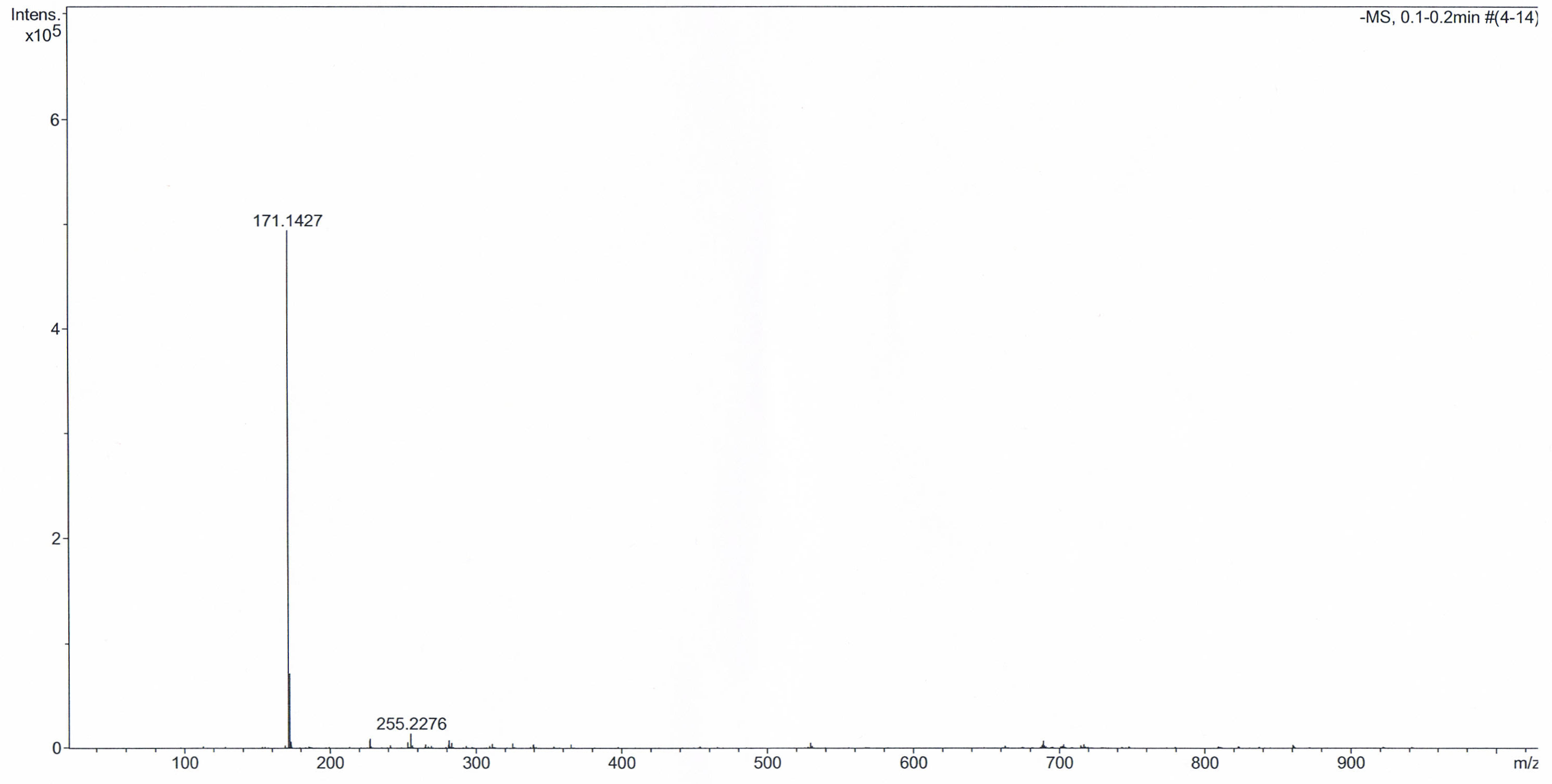

Supplement: Figure S4 — ESI/MS spectrum of capric acid (C10:0) standard. (0.31 MB PNG) [file pone.0012050.s004.png]
